# Predicting mortality risk for preterm infants using random forest

**DOI:** 10.1038/s41598-021-86748-4

**Published:** 2021-03-31

**Authors:** Jennifer Lee, Jinjin Cai, Fuhai Li, Zachary A. Vesoulis

**Affiliations:** 1 Washington University School of Medicine, St. Louis, USA; 2 Division of Biostatistics, Washington University School of Medicine, St. Louis, USA; 3 Institute for Informatics, Washington University, St. Louis, USA; 4 Department of Pediatrics, Division of Newborn Medicine, Washington University School of Medicine, 1 Children’s Place, Box 8116, St. Louis, MO 63110 USA

**Keywords:** Biomarkers, Risk factors, Paediatric research, Machine learning, Predictive medicine

## Abstract

Mortality is an unfortunately common outcome of extremely and very preterm birth. Existing clinical prediction models capture mortality risk at birth but fail to account for the remainder of the hospital course. Infants born < 32 weeks gestation with complete physiologic and clinical data were included in this retrospective study. Mortality risk was quantified by conventional means (clinical factors) using the CRIB-II score and the optimal logistic regression model. A random forest (RF) model was trained using a subset of the cohort, labeling data within 6 h of death as “worry.” The model was then tested using the remaining infants. A total of 275 infants were included in the study with a mean gestational age of 27 weeks, mean birth weight of 929 g, 49% female, and a mortality rate of 21%. The CRIB-II and logistic regression models had acceptable performance with sensitivities of 71% and 80% AUC scores of 0.78 and 0.84, respectively. The RF model had superior performance with a sensitivity of 88% and an AUC of 0.93. A random forest model which incorporates fixed clinical factors with the influence of aberrancies in subsequent physiology has superior performance for mortality prediction compared to conventional models.

## Introduction

The WHO estimates that 15 million babies are born prematurely each year^1^, a problem which continues to increase over time^2,3^. Premature birth arises from a myriad of causes including increasing maternal age, multiple gestation pregnancy, and increased frequency of medical co-morbidities such as hypertension and diabetes. Very low birth weight (VLBW) infants, those born before 32 weeks gestational age (GA) and weighing less than 1500 g, are at significant risk for mortality, ranging between 10 and 70%, depending on the GA at birth^4,5^. Death frequently occurs as the result of an acute process such as respiratory failure, sepsis, or intraventricular hemorrhage^6,7^.

There are several published methods for prediction of mortality including the NICHD Outcomes calculator^5,8^, the Clinical Risk for Infants and Babies (CRIB-II) score^9^, and the SNAPPE-II score^10^. While each of these methods were produced using large population datasets, they rely on fixed perinatal factors (such as gestational age and birth weight) and fail to account for dynamic changes in the infant over the course of hospitalization. While there is value in an instantaneous estimate of mortality around the time of neonatal ICU (NICU) admission, this calculation is of progressively lower relevance as the hospitalization progresses. Models that account for postnatal developments have also been proposed^11^, but these too utilize only static factors, measured at various timepoints during the course of hospitalization. Risk estimates are thus only updated on an intermittent basis, at best.

The ideal approach for mortality prediction would supply continuous risk assessment, incorporate clinical data across multiple domains (e.g. demographic, clinical, and vital sign data), and be continuously updated as the patient’s condition changes. Neonatal intensive care has evolved dramatically over the last few decades, with a significant component of that evolution being the deployment of electronic physiological monitoring systems. NICUs have transitioned from using unreliable cardiac monitors and spot-check pulse oximetry to continuous, high-quality measurement of heart rate (HR), blood pressure (BP), pulse oximetry (SpO2), and more. This transition has also resulted in a great deal more data than can feasibly be manually interpreted by a single provider. Although patient monitors provide the ability to set alarms when vital signs depart from expected ranges, false positive and negative alarms are a significant problem and frequently lead to alarm fatigue^12^. Thus, real-time complete situational awareness of evolving patient conditions across the entire unit is an often challenging, and frequently impossible, task.

An automated method for predicting impending mortality, with a sufficiently large lead-time, may improve provider focus on decompensating patients and facilitate opportunities for earlier intervention, thereby changing outcomes. There is precedent for this approach; the HeRo^13,14^ and nSOFA^15^ scores utilize changes in heart rate variability or data extracted from the electronic medical record (EMR), respectively, to provide a continuous estimate of sepsis risk. While both methods are valuable and have been validated, they only provide information pertaining to risk of sepsis and not the remaining sources of mortality risk.

In this study, we describe an interpretable machine learning (ML) based approach for continuous quantification of mortality risk in a cohort of very preterm infants. Specifically, a random forest classifier is trained to predict impending mortality based on (1) static demographic and clinical factors, which describe an infant’s a priori risk, and (2) dynamic vital sign data, which provides insight into the dynamic changes that modify this initial risk. Model performance is evaluated via four key metrics: accuracy, sensitivity, specificity, and area under the receiver-operator characteristic (ROC) curve, and via comparison against an existing, validated clinical mortality risk score (CRIB-II) as well as a baseline logistic regression model trained only on static demographic/clinical factors.

## Methods

### Cohort selection

All infants admitted to the NICU at St. Louis Children’s Hospital, a level IV NICU serving urban, suburban, and rural populations have vital sign data prospectively archived into a database (BedMaster EX, Excel Medical, Jupiter, FL). Inclusion criteria for this study were birth before 32 weeks completed gestation and at least 80 h of recorded vital sign data. Infants meeting these criteria were retrospectively identified and extracted from the archive. The study protocol was reviewed and approved by the Washington University IRB, a part of the Human Research Protection Office, and waived the need for informed consent pursuant to 45 CFR § 46.116 section F. All research methods were performed in accordance with relevant local, state, and federal guidelines and regulations.

### Vital sign measurements

All infants were monitored using Philips Intellivue MP70 or MX800 patient monitors (Philips Medical, Andover, MA) and signals were recorded as the time-integrated mean with a sampling rate of 1 Hz. All infants had a common set of vital sign parameters—heart rate (HR), respiratory rate (RR), and pulse oximetry (SpO2). Some infants had invasive arterial lines, which provided three additional signals (ART-S, ART-M, ART-D; arterial systolic, mean, and diastolic blood pressure). Infants without invasive arterial lines had non-invasive blood pressure measurement (NIBP-S, NIBP-M, NIBP-D; non-invasive systolic, mean, and diastolic blood pressure). Arterial pressures were sampled at the same 1 Hz rate as the other vital signs, whereas NIBP measurements were made sporadically, but at least once every twelve hours per clinical guidelines.

While vital sign data provide an important dynamic component to the prediction of mortality, they should be viewed as modifiers of the infant’s a priori risk. In addition to the vital signs, four highly relevant demographic/clinical factors were included (gestational age [completed weeks], sex, race, and birth weight).

### Data pre-processing

Missing or out-of-range values are common in vital sign data derived from a clinical setting. Viable ranges for each vital sign were defined as follows: HR [0, 250], RR [0, 120], SpO2 [0, 100], BP [10, 90]. Missing or out-of-range values were first each replaced with not-a-number (NaN). NaN entries for each vital sign were then replaced via imputation using mean values for that variable across all training or testing data, respectively.

### Feature extraction

Vital sign data was originally sampled at 1 Hz and was downsampled to every ten seconds for model training and testing. Noise from motion artifact, poor sensor contact, or interference from other devices is a common and unavoidable part of physiologic data collection in the NICU environment. To reduce the influence of these brief outlier values, dynamic variables were incorporated into the model as rolling means, standard deviations, and absolute z-scores. We computed each of these over two different sizes of windows (5 min and 30 min). In total, 34 features were used for random forest model training and testing (see Supplementary Table 1).

### Random forest classifier

To assess mortality risk, the Scikit-learn^16^ implementation of the random forest algorithm was used to develop a model to predict impending mortality (within the next six hours). Random forest classification involves growing several trees using bootstrapped samples from training data. Majority voting is then used to determine the terminal node (“worry” or “don’t worry”) to which each data point should be assigned. Notably, randomness is added at each node by choosing a random sample of predictors to consider for the split. The end result is a forest constructed from randomly selected cases and randomly selected predictors.

The per-timepoint random forest model was trained, validated, and tested using data from 275 infants, for which vital sign and demographic/clinical information were available. Training and validation data consisted of data from 206 randomly selected infants (~ 75% of the total dataset), which yielded a total of 31,287,801 data points. Each data point represents an infant at a given timestamp—*i.e.*, is a “snapshot” of an infant’s dynamic variables (HR, BP, RR, SpO2) and static variables (gestational age, sex, race, and birth weight)—and was either labeled as “worry” or “don’t worry.” A data point was labeled as “worry” if the infant died within six hours of that data point’s timestamp. “Worry” and “don’t worry” labels were assigned values of 1 and 0, respectively.

After evaluation of model performance across a range of timeframes, the “worry” timeframe of six hours was chosen as a compromise between model performance and reasonable window of opportunity for clinical intervention. Additionally, as mortality is relatively rare (approximately 10% of infants born before 32 weeks gestation), there were many more data points labeled “don’t worry” as compared to those labeled “worry” (31,190,601 “don’t worry” and 97,200 “worry,” respectively). For training only, the “don’t worry” data points were randomly subsampled to match the number of the “worry” data points, thereby accounting for this class imbalance.

An important distinction from existing models, such as the CRIB-II score, is that the per-timepoint random forest model outputs several predictions for a given infant throughout their NICU stay—that is, one prediction per timepoint, rather than one per infant. For the purpose of comparison with the thresholded CRIB-II scores and the baseline logistic regression model, the per-timepoint random forest classifier results were modified to output a single prediction per infant (“worry” or “don’t worry”) by taking the majority prediction made over the infant’s last six hours.

### Model parameters

Parameters of the random forest model (number of estimators and maximum tree depth) were subjected to a grid search with k-fold cross validation (k = 5) to find optimal values that increased robustness and prevented overfitting. Specifically, we selected four possibilities for max_depth (2, 4, 8, 12), and four possibilities for n_estimators (25, 50, 100, 200); these values represent typically accepted values for random forest models. Each possible combination of those two parameters was used to build a specific random forest model.

We then evaluated each model with k-fold cross validation. In this approach, the entire cohort is divided into k equal parts. K-1 sets are used for training while the remaining set is set aside for testing. The algorithm is trained and tested k times (k = 5 in this case). A result is generated for each cross-validated model by taking the average obtained on each set.

The final parameters (number of estimators = 100, maximum tree depth = 4) were chosen based on an evaluation of model accuracy (Supplementary Fig. 1), sensitivity, specificity, and AUC score. The model was then tested on data from the remaining 69 infants (~ 25% of the total dataset), which yielded a total of 8,845,659 data points. No correction was made for the class imbalance in testing data since that imbalance will inevitably exist in real-time datasets.

### Logistic regression model

Logistic regression classification is a machine learning algorithm that uses as dependent variable the log of odds and predicts outcomes with a logit function. It is typically used as the baseline comparison for any binary classification problem, given that it is not only relatively straightforward to implement, but also capable of good performance. The logistic regression model was trained on static demographic/clinical data only and outputted a single prediction (“worry” or “don’t worry”) for each infant. A “worry” prediction was considered correct if the infant eventually died.

### Model evaluation metrics

To evaluate the performance of each model, the logistic regression and random forest models were compared to the CRIB-II score, which utilizes only static demographic/clinical variables, akin to typical clinical practice today. Given that CRIB-II scores ≥ 11 are significantly associated with mortality^17,18^, infants with CRIB scores at or beyond 11 were labeled as “worry.” As with the logistic regression model, a “worry” prediction was considered correct if the infant eventually died.

The accuracy, sensitivity, and specificity of each model were calculated to compare and evaluate model performance. Models were additionally evaluated via calculation of the area under the curve (AUC) score in ROC analysis.

## Results

### Cohort description

A total of 275 infants were included in the study with a mean gestational age of 27 weeks, mean birth weight of 929 g, 49% female, and a mortality rate of 21%. Complete demographic and clinical details of the cohort can be found in Table 1.

**Table 1:** Patient demographics.

	All (n = 275)	Survived (n = 216)	Died (n = 59)
**Gestational age, completed weeks**			
Mean (SD)	27 (2)	27 (2)	25 (2)
Range	22—31	22—31	22—31
**Birth weight, grams**			
Mean (SD)	929 (274)	991 (257)	704 (207)
Range	360—1520	360—1520	410—1390
**Sex**			
Male, n (%)	139 (50.55%)	111 (51.39%)	28 (47.46%)
Female, n (%)	136 (49.45%)	105 (48.61%)	31 (52.54%)
**Race**			
White or Caucasian, n (%)	151 (54.91%)	118 (54.63%)	33 (55.93%)
Black or African American, n (%)	110 (40.00%)	90 (41.67%)	20 (33.90%)
Asian, n (%)	3 (1.09%)	1 (0.46%)	2 (3.39%)
Unknown/Not Reported, n (%)	11 (4.00%)	7 (3.24%)	4 (6.78%)

### Mortality prediction

The original per-timepoint random forest model, which makes a prediction for every timepoint (*i.e.*, many predictions per infant), performed well, with an overall accuracy of 80%, a balanced sensitivity and specificity of 0.79 and 0.80, respectively, and an AUC score of 0.88 (see Table 2, Supplementary Fig. 2).

**Table 2:** Model metrics for per-timepoint random forest.

	Per-timepoint random forest with dynamic and static variables
Accuracy	0.80
Sensitivity	0.79
Specificity	0.80
AUC score	0.88

**Figure 1:**
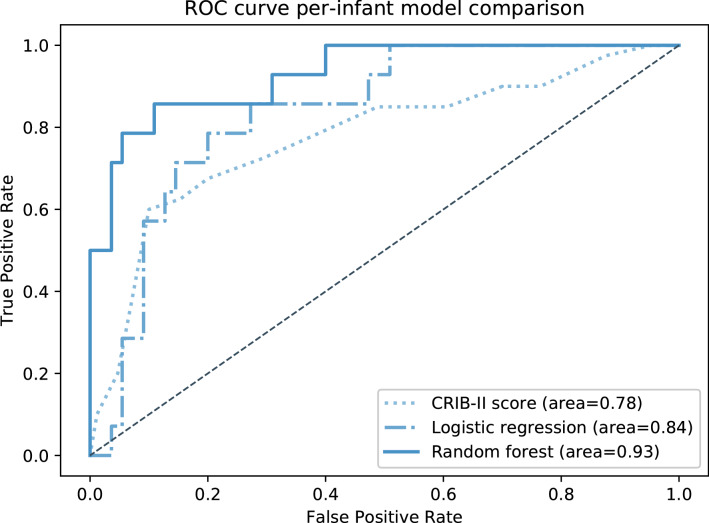
ROC curves for per-infant model comparison. Comparison of receiver operating characteristic (ROC) curves for the CRIB-II score, logistic regression model, and per-infant random forest model. Each of these three approaches makes a single prediction (“worry” or “don’t worry”) per infant, where “worry” indicates impending mortality.

As is the case with other previously published mortality prediction algorithms, the CRIB-II scores and logistic regression model using only static demographic/clinical variables (gestational age, sex, race, and birth weight) both had acceptable performance, with a moderately high accuracy of 71% and 80% respectively, and excellent specificity, albeit at the cost of sensitivity. In addition to the poor sensitivity, the AUC scores of 0.78 and 0.84 were only modest to good (see Table 3, Fig. 1).

**Table 3:** Comparison of per-infant model metrics.

	CRIB-II score with threshold of 11	Logistic regression with static variables only	Per-infant random forest with dynamic and static variables
Accuracy	0.71	0.80	0.88
Sensitivity	0.73	0.21	0.86
Specificity	0.71	0.95	0.89
AUC score	0.78	0.84	0.93

The modified per-infant random forest model, which makes a single prediction per infant by taking the majority prediction made over the infant’s last six hours, performed markedly better than not only the thresholded CRIB-II scores and the baseline logistic regression model, but the per-timepoint random forest model as well. Performance far exceeded that of other models, with an accuracy of 88% and a much higher AUC score of 0.93 (see Table 3, Fig. 1, Supplementary Fig. 3).

**Figure 2:**
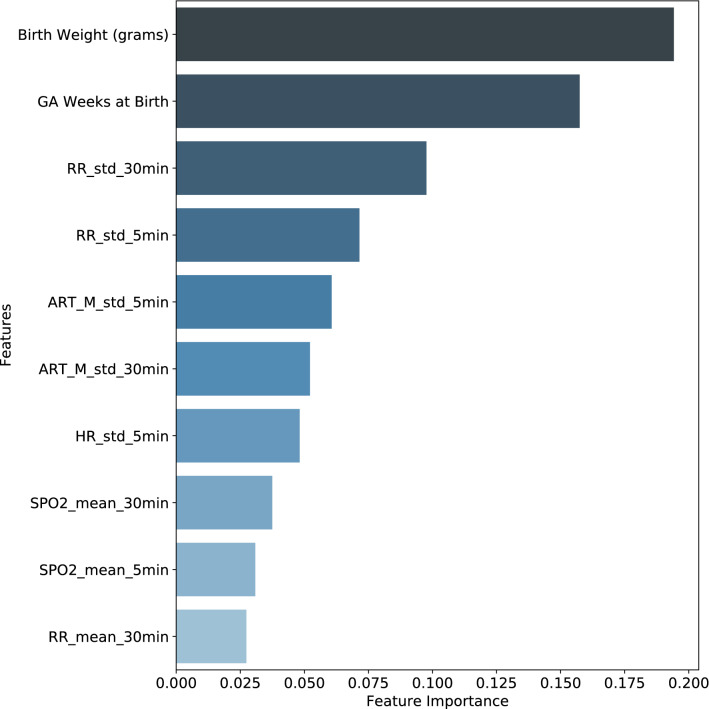
Per-timepoint random forest top ten features ranked by importance. Ten most important features of the per-timepoint random forest model, ranked according to the mean decrease in Gini impurity.

The two most important features in the per-timepoint random forest model were both static demographic/clinical variables—birth weight and gestational age (Fig. 2). These were followed in importance by several dynamic vital sign variables, the most impactful of which was respiratory rate.

## Discussion

Accurate and timely prediction of mortality in the NICU offers an alternative route to alerting providers of impending catastrophe and opens a window of opportunity for intervention to change course. By taking both key clinical factors and streaming vital sign data into account, we have trained and validated an interpretable machine learning model which predicts impending mortality with good performance.

Importantly, the performance of these models is enhanced by the use of clinical risk factors (gestational age, sex, race, and birth weight). A true representation of an infant’s risk for mortality comes from not only from non-modifiable demographic and perinatal factors, but also the dynamic events of the NICU course which might modify this initial risk. This composite approach offers a more personalized estimate of mortality risk, one which changes with the patient.

It is important to note that while race is largely a social construct, it is frequently a proxy for increased risk of adverse outcome, the result of inequality in access to health care. The inclusion of race as a factor in any predictive model is a gross oversimplification of several socioeconomic and behavioral characteristics, many of which are outside the patient’s control. That being said, risk models are incomplete without consideration of race. Furthermore, the cohort used in this study is relatively balanced demographically (see Table 1), so there are fewer concerns about training on small numbers.

The best performing features provide further evidence to support the value of mixed data types. Not surprisingly, birth weight and gestational age are major drivers of mortality risk. There is a significant body of evidence to support the role of each of these two factors in predicting mortality. One recent study of more than 8000 infants readily demonstrates the rapid decline in mortality risk from more than 40% at 23 weeks gestation to little more than one percent by 30 weeks^19^. Low birth weight is an independent determinant of mortality risk, even when controlling for demographic and inter-center factors^20^.

**Figure 3:**
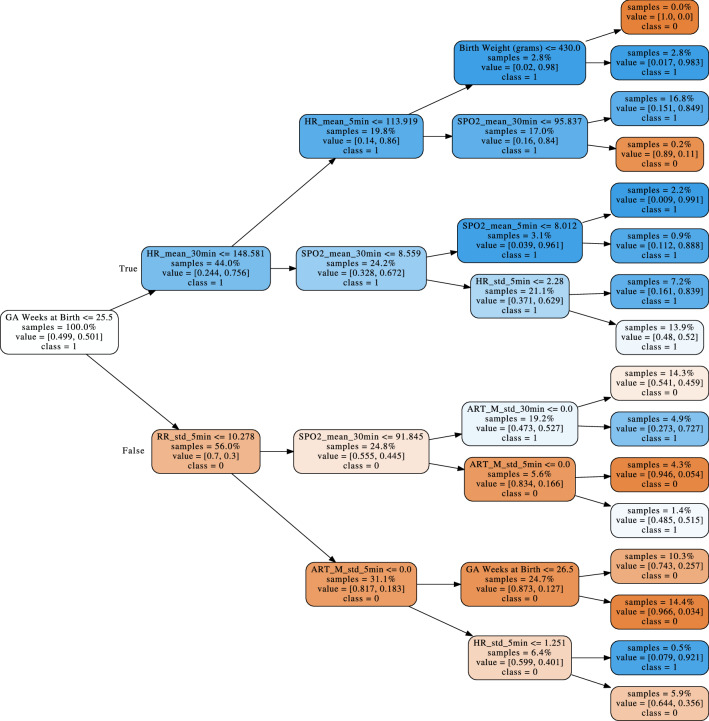
Example decision tree from per-timepoint random forest model. One of the decision trees used in the per-timepoint random forest model. Orange indicates a majority prediction of “don’t worry,” whereas blue indicates a majority prediction of “worry.” A more intense coloring designates a lower Gini impurity, which in turn indicates a lower likelihood of misclassification.

Vital sign features, especially the respiratory rate and mean arterial blood pressure, also rank highly in terms of feature importance for mortality prediction, as illustrated by their presence in the top levels of the random forest model trees (see Fig. 3). A recent analysis of 4500 VLBW infants across 10 different NICUs in Australia revealed that the most common causes of mortality are intraventricular hemorrhage (IVH), acute respiratory illness, and sepsis^7^. Not surprisingly, these conditions are marked by apnea, hypotension, and bradycardia, all of which are captured by this model.

Although the focus of this analysis is on the added value of continuous vital signs, the mere presence of some vital sign types may be an indicator of severity of illness (and risk of mortality). For example, in this study the presence of arterial blood pressure may indirectly indicate a critically ill infant, as current NICU practice is to minimize central line exposure where possible. In order to capture this information, a central arterial line must be placed (generally via an umbilical artery). There are significant risks associated with placement and maintenance of this line—thus, the presence of this data suggests that the severity of critical illness weighs in favor of the invasive monitoring.

**Figure 4:**
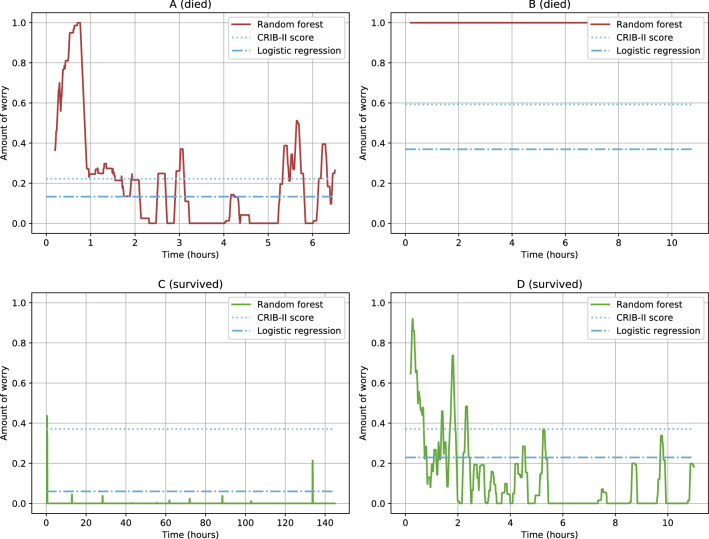
Per-timepoint random forest predictions over time for randomly selected infants. Per-timepoint forest model predictions plotted over time for four randomly selected infants (labelled A-D). Each graph plots the rolling average of predictions (per timepoint) over one hour. Values range between 0.0 (“don’t worry”) and 1.0 (“worry”) (*i.e.*, higher values indicate that more “worry” predictions were made in the past hour). For comparison, the a priori CRIB-II score predictions (represented as a proportion of the maximum CRIB-II score, 27) and logistic regression probability estimates are also plotted.

While the intent of this project is to develop “just in time” prediction of mortality and enable timely intervention, additional insight may be obtained from longitudinal analysis of predictions. To illustrate this point, we review the predictions made for two randomly selected infants who died and two randomly selected infants who survived (see Fig. 4). For the two infants that died (infants A and B), the random forest model repeatedly or consistently predicted “worry” throughout their NICU stay, in line with their health trajectories and eventual demise. For one of the two infants that survived (infant C), the random forest model consistently predicted “don’t worry,” again in line with their health trajectory and eventual discharge to home. Conversely, for infant D, there were a high number of “worry” predictions. In other words, infant D ended up surviving, but raised many alerts along the way. A quick chart review reveals a clinical vignette that is indeed extremely worrying, despite the outcome of survival. Infant D was quite sick throughout his stay, with diagnoses of cystic periventricular leukomalacia, septicemia, and significant lung disease.

These observations raise two important questions which should be investigated in future studies. The persistence of “worry” prediction for infants who ultimately died suggest that this model, or a future derivative, may be able to predict mortality beyond the six-hour threshold used in this study. Second, the presence of “false positives,” sick infants who ultimately do not die, suggest that a more granular model might be developed with three outcomes of low mortality risk, medium mortality risk/high risk of complications, and high mortality risk.

There are several real and possible limitations of this study. First, like all machine learning applications, the larger the training dataset, the greater accuracy can be achieved. With the current sample, we have demonstrated that we can achieve prediction accuracies in excess of current clinical-only models. Future studies of even larger populations should be considered to further improve the model. Second, site-specific differences (*e.g.* clinical practices and patient populations) may prevent generalization of the results. External validation should be a high priority for future studies to evaluate the durability of these findings. Third, we have used vital signs commonly obtained in a NICU setting. Other biosignals, such as cerebral oximetry, transcutaneous CO2 measurement, or streaming data from other medical devices such as ventilators may provide valuable additional information and should be incorporated into future lines of investigation. Finally, we have chosen an arbitrary time point of six hours before death as a compromise between model performance and a sufficient warning period. Depending on the goals and context of different providers, other time intervals should be investigated.

In this study, we have demonstrated that dynamic vital sign and fixed clinical/demographic factors can be used in a machine learning model to predict imminent death in a cohort of preterm infants. Risk of adverse outcome in the NICU is driven by inherent, fixed factors, but this risk is dynamically modified by the events of hospitalization. The development of tools which incorporate all elements of risk and provide a continuously updating assessment will be essential for implementation of truly personalized care and potentially life-saving measures.

## Supplementary Information

Supplementary Information 1.

## Data Availability

Privacy rules preclude release of the datasets generated and/or analyzed during this study.
