# Supplementary material for: Predicting mortality risk for preterm infants using random forest
Source: Sci Rep. 2021 Mar 31;11:7308. doi: 10.1038/s41598-021-86748-4 (PMC8012581; doi:10.1038/s41598-021-86748-4)
Supplement: Supplementary file 1 — Supplementary Information 1. [file 41598_2021_86748_MOESM1_ESM.docx]

**Supplementary File**

**Title:** Predicting mortality risk for preterm infants using random forest

**Authors:** Jennifer Lee, Jinjin Cai, Fuhai Li, Zachary A. Vesoulis

**Supplementary Tables**

**Supplementary Table 1:** Random forest features

|  | **Raw** | **Mean** | | **Standard deviation** | | **Absolute z-score** | |
| --- | --- | --- | --- | --- | --- | --- | --- |
| Window size (for rolling features) |  | 5 min | 30 min | 5 min | 30 min | 5 min | 30 min |
| **Heart rate** |  | **X** | **X** | **X** | **X** | **X** | **X** |
| **Blood pressure**, (arterial mean) |  | **X** | **X** | **X** | **X** | **X** | **X** |
| **Blood pressure**, (non-invasive mean) |  | **X** | **X** | **X** | **X** | **X** | **X** |
| **Respiratory rate** |  | **X** | **X** | **X** | **X** | **X** | **X** |
| **Oxygen saturation** |  | **X** | **X** | **X** | **X** | **X** | **X** |
| **Gestational age** | **X** |  |  |  |  |  |  |
| **Sex** | **X** |  |  |  |  |  |  |
| **Race** | **X** |  |  |  |  |  |  |
| **Birth weight** | **X** |  |  |  |  |  |  |

**Supplementary Figures**

**Supplementary Figure 1:** Parameter Grid Search with k-Fold Cross Validation**
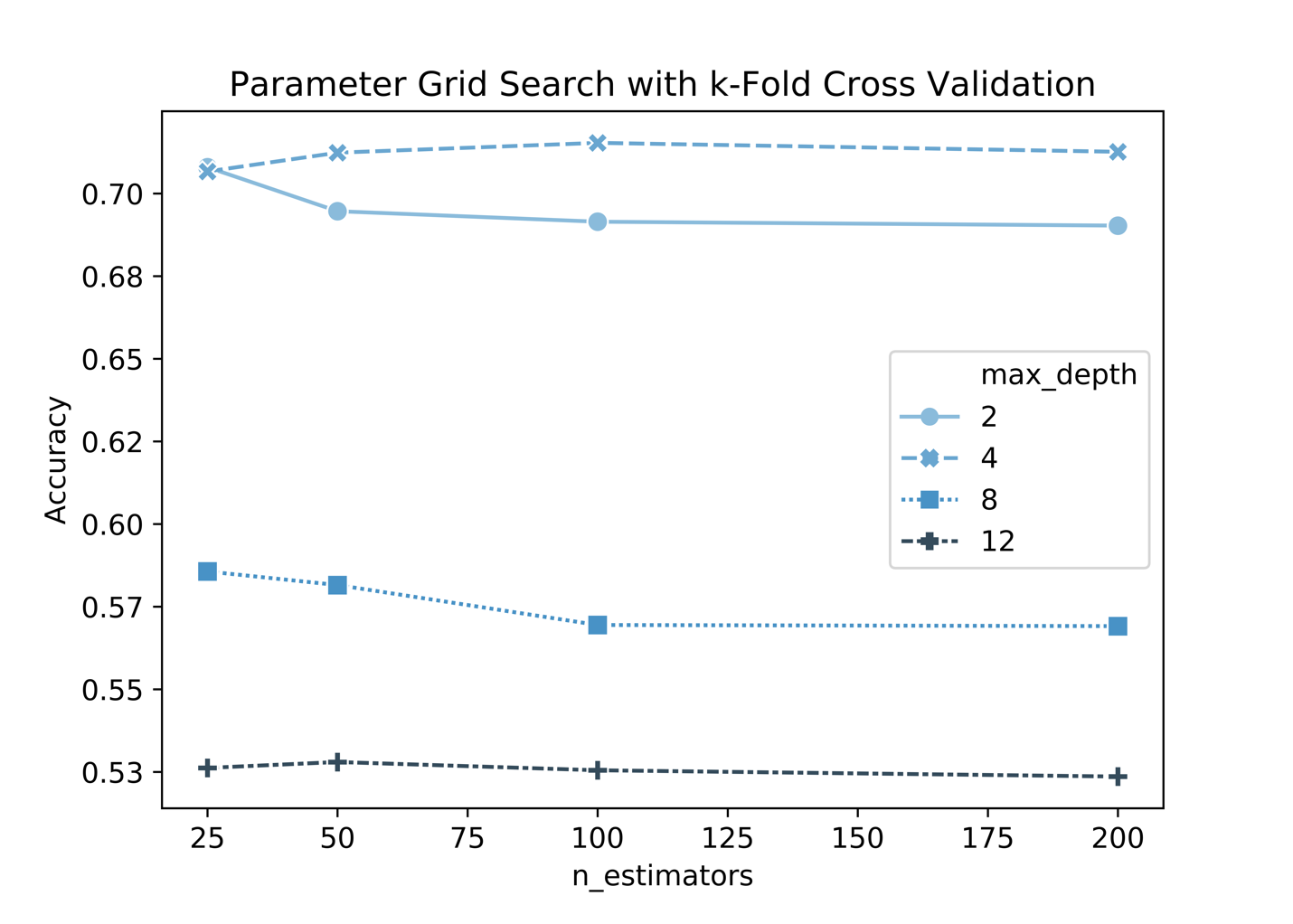
**

**Caption:** Results of grid search with k-fold cross validation to select random forest model parameters (number of estimators and maximum tree depth).

**Supplementary Figure 2:** ROC curve for per-timepoint random forest

**Caption:** ROC curve for the per-timepoint random forest model, which makes a single prediction (“worry” or “don’t worry”) per timepoint.

**Supplementary Figure 3:** Confusion matrices for per-timepoint and per-infant random forest models

**Caption:** Confusion matrices for the per-timepoint and per-infant random forest models. TP indicates the number of true positives, FP the number of false positives, FN the number of false negatives, and TN the number of true negatives.
